# A Case of Rhino-Lithiasis

**Published:** 1886-02

**Authors:** O. Chiari, W. S. Renner

**Affiliations:** 258 Franklin street, Buffalo, N. Y.


### Translations.

### A Case of Rhino-Lithiasis.

By Dr. O. Chiari.

Translated from the *Wiener Medicinische Wochenschrift*, by W. S. Renner, M. D.

I. A young lady, Mrs. S., on the recommendation of Professor Nothnagel, came to consult me on the 19th of August. She complained of obstruction of the right side of the nose, and of a discharge from the same, which had persisted for more than ten years. In addition to this, she had suffered, for the same length of time, from severe periodical headache, which especially attacked the occipital region, and from indigestion, which had been much relieved by the water-cure at Karlsbad, but the headache remained unchanged. By anterior rhinoscopy, the lower turbinated bone appeared considerably swollen and reddened, and the very narrow passage between the lower turbinated bone and the septum filled with pus. After the removal of the pus, I entered the nose by means of a sound, and came, in the middle of the inferior nasal passage, upon a rough, bony, hard-feeling body, which also extended into the middle nasal passage, where it appeared to be much thinner. On compressing the lower turbinated bone with a spatula-shaped sound, I was able to see that this body had a gray and uneven surface; the posterior margin of this body could not be reached with the sound. Posterior rhinoscopy showed only a slight swelling and redness of the lower and middle turbinated bones, but no part of the foreign body. I endeavored to seize and extract it with a pair of surgeon's tweezers; but, as it did not yield to the traction, I finally took a pair of slender polypus-forceps. With these I broke off a part, which proved to be a concretion of brownish-yellow color, and broke into small pieces by the use of some force. After I had continued to lessen the size of the body for some days with strong polypus-forceps, I was able to move it, but could neither pull it out nor shove it back into the throat. Fortunately, I finally secured some of its projections, crushed them with considerable force, and extracted it whole, with but slight bleeding. It proved to be a rhomboidal body 2 ctm. long, 1—1½ ctm. broad, and ½—1 ctm. thick, and its surface was irregularly uneven brown, and thickly covered with mucus. The broken planes of this hard body were white and uneven. On scratching away the superficial stratum in places with a pin, I came upon a metal resistance, which soon proved to be a round, flat metal button about 13 mm. in diameter. It was enclosed on all sides by the concretion, and was evidently the cause of the formation of the calculus. Professor Muthner kindly examined some of the pieces which were broken off. They consisted chiefly of the carbonates and phosphates of lime and magnesia, with a small amount of organic matter. The whole concretion, including the small pieces, weighed about 3½ grammes. Distinct concentric layers could he recognized on the cross section.

II. This was evidently a rhinolith which had formed about a foreign body. The patient had no idea when the button was introduced into the nose. But we naturally conclude, from the headache and nasal discharge lasting over ten years, that the patient, when a child, had stuck it in her nose and afterwards forgot about it.

III. On examining the nose a few days after the extraction, only a few excoriated spots were to be seen on the mucous membrane of the septum and lower turbinated bone. The patient had partly lost the headache, and the discharge had quite disappeared, and she only complained that too much air passed through the nose, producing dryness of the throat. A perusal of the literature of the subject will show that my case healed in the same manner as most of those which have been described. I have been able to discover forty-nine reported cases.

IV. How do nasal calculi originate? They can frequently be referred to a foreign body in the nose. Mackenzie, at least, holds this opinion. Demarquay noticed the same thing in the fifteen cases which are reported in his work; of the remaining thirty-four (with mine, thirty-five) cases, twenty-one were proved to have foreign bodies as their centers, eight were without, in three it was not mentioned, in one (Nourse) a bone was probably the center, and in the last, Störk could not make out whether the center was composed of metamorphosed bone or of a piece of rhino lithic lime. Brown discovered oxide of iron in the ashes of the soft kernel of a rhinolith. As the patient was a workman, it is not improbable that at some time a splinter of iron entered the nose, and became the source of the development of this stone, which, with the mucus about it, afterwards formed the soft kernel. Likewise, in Mackenzie's two cases, no foreign bodies could be discovered as the nuclei; still, Mackenzie himself says that in the second case such a thing might easily have escaped observation, as he was compelled to crush the stone several times, on account of its size. In Verneuil's case the center consisted of a cheese-like mass composed of epithelial cells and pus corpuscles, and in Guttceit's case of a soft, greenish substance.

V. In some of these thirteen cases, the absence of a foreign body is doubtful. Mackenzie says, on this point, that a foreign body may have been the cause of the formation of a calculus, and afterwards gradually became a soft mass. At least a foreign body appears to be necessary to the formation of a nasal calculus. Other causes seldom act; but chronic catarrh, constriction and obstruction of the nose are mentioned as such (Brown). Both these conditions favor the deposit of small clumps of mucus and blood clots, which may act as centers for the deposit of lime salts.

VI. Nasal calculi are usually single, but cases are known where more than one have occurred (Axmann, Demarquay, Mackenzie). They sometimes attain a considerable size (to that of a pigeon's egg, Verneuil), and a weight up to 15 grammes (Brown), and can remain in the nose for years, viz., twenty-one years (Jacquemart), and forty years (Bettmann). They consist of the carbonates and phosphates of lime and magnesia, with traces of sodium salts. Traces of albumen, mucus fibrin, and fat also occur. They are usually found in the lower nasal passages, and are sometimes partly covered by a hypertrophy of the mucous membrane, and may cause the turbinated bones to be excoriated or ulcerated; they may even cause necrosis of the turbinated bones (Hering). A muco-purulent discharge, which often has a bad smell, and obstruction of the corresponding side of the nose, occur almost always; and very frequently headache and other nervous disturbances are met with. The external form of the nose changes but slightly.

VII. In making a diagnosis, you must take into consideration the fact that the calculus is usually covered with swollen mucous membrane, and, consequently, that the examination should always be made with the help of a sound. The recognition of a hard, uneven body, however, does not signify, for an ossified tumor and a chalky deposit in the mucous membrane

(Virchow) produce the same sensation, when examined with a sound. We can, however, exclude these diseases, by taking into consideration that a stone may exist for years without producing deformity of the nose and necrosis, and that it grows very slowly, while necrosis of the bone usually has its origin in syphilis, and, like neoplasms, soon changes the form of the nose. And, in addition, calculi are usually somewhat movable, which is not the case with osteomæ and similar formations, and, finally, we must take into consideration the history of the case, but just here we can seldom learn anything definite; especially must our attention be drawn to the fact that children often stick foreign bodies into the nose, and either conceal the fact through fear of punishment, or really forget about it.

VIII. In any case the treatment is the same, especially where we are not certain whether the object is a foreign body or a calculus. If the stone is small and easily movable, it can be removed by introducing a sound with the end shaped like an ear-pick beyond it and drawing it forward. Or you may try to wash it forward by irrigation from the opposite side of the nose, and finally you may extract it with a pair of tweezers. Shoving the stone back into the throat, while keeping watch over the entrance into the larynx, has been practiced (Hering). Very large stones must first be lessened in size before they can be extracted. This has sometimes been accomplished by irrigating with lukewarm water for some time (Störk). They are generally so hard that they require to be crushed, be it by means of strong polypus-forceps or a lithotrite (Verneuil, Mackenzie). Stockton perforated the concretion in one case by means of a drill. When the stone is finally removed, complete healing soon follows, but the affected side of the nose always remains somewhat more spacious.

(The translator has omitted the list of authors consulted).

258 Franklin street, Buffalo, N. Y.

